# The Cholera

**Published:** 1849-01

**Authors:** 


### ARTICLE I.

### THE CHOLERA.

This dreaded malady, as is well known, is gradually, but steadily and surely making its advances towards the West; and, from the last accounts, we have reason to believe that it has already reached this country.

In nearly all our recent exchanges, we find facts and remarks upon this subject, more or less extensive.

It seems to be generally admitted, that its malignity is in proportion to want of salubrity, and to the extent to which influences exist in any locality, favorable to the production of typhoid and other epidemics, and that the habitually intemperate and profligate are the ones most frequently attacked, "so much so, that it is a very rare thing for the intemperate to escape; generally speaking, it is almost as rare for the temperate and uniformly prudent to be attacked."

As might have been expected, members of the profession, both in this country and in Europe, are constantly taxing their ingenuity to find facts and devise theories to account for its sudden and mysterious appearance in different localities, for the symptoms during an attack, and for the various contradictory statements concerning the effects of different kinds of treatment.

The theory, to account for the phenomena presented by it, which seems to be popular with the greatest number, is what may be called the electrical, which supposes it to depend upon electric or galvanic influences.

It is well known that the chemical and other changes constantly going on upon the surface of the earth in the atmosphere, and in our own and the bodies of other animals, are necessarily attended with the development of electricity, or more properly speaking, they give rise to currents as often as the electrical equilibrium is disturbed, in consequence of the variety in the extent, and nature of the changes in different localities, and under different circumstances. That this subtle agent has more to do in modifying vital action, both in health and disease than had been formerly supposed, is not to be doubted, since all recent investigations, both in vegetable and and animal physiology, tend to show that the organic change, upon which the life of a plant or animal depends, is in a great measure dependent upou its influence. This being the case, we have only to consider that the intensity of the earth's electricity at different points is constantly varying, as is shown by the variation in the magnetic needle; and 'that local disturbances and currents must be constantly produced by evaporation and chemical changes constantly going on in wet, marshy districts in the country, and in the damp, crowded, and filthy streets in large cities; and it appears reasonable, to say the least, to admit that the electrical changes thus constantly occurring must have an influence in modifying, if not in producing disease.

In the London Lancet, for September, we find a long article upon this subject, by Sir James Murray; in which he states that numerous experiments and observations, made during the prevalence of cholera in Dublin, in 1832, force upon him the conviction that not only this disease, but nearly all epidemics are dependant upon the above named causes; and, in accordance with this view, it is proposed by him to insulate buildings, especially such as are occupied for sleeping, as a preventative to this class of diseases.

He supposes that such diseases are consequent upon molecular changes, produced either by currents of electricity passing through, or by an excess or deficiency of this fluid in the body, and that the proper preventative, therefore, is to use, as far as is practicable, non-conducting substances for building material and clothing. H.

